# Error in Additional Contributions

**DOI:** 10.1001/jamahealthforum.2023.4488

**Published:** 2023-12-01

The Viewpoint titled “The CMS Strategy to Promote Equity in Quality and Value Programs,”^1^ published online October 20, 2023, included a misspelling in the Additional Contributions acknowledgment. The name Lee A. Fleischer, MD, was corrected to Lee A. Fleisher, MD. This article has been corrected online.
